# Repeat induced abortion and associated factors among women seeking abortion care services at Debre Markos town health institutions, Amhara regional state, Ethiopia, 2017

**DOI:** 10.1186/s13104-020-4904-x

**Published:** 2020-01-28

**Authors:** Mahlet Imana Waktola, Dawit Gebeyehu Mekonen, Tewodros Seyoum Nigussie, Endeshaw Adimasu Cherkose, Addisu Taye Abate

**Affiliations:** 1 College of Health, Department of Midwifery, Mizan Tepi University, Mizan Tepi, Ethiopia; 2 College of Medicine and Health Sciences, School of Midwifery, University of Gondar, Gondar, Ethiopia; 3 College of Medicine and Health Sciences, School of Nursing, Department of Medical Nursing, University of Gondar, Gondar, Ethiopia

**Keywords:** Repeat induced abortion, Cross–sectional study, Debre Markos town

## Abstract

**Objective:**

The aim of this study was to assess the proportion and determinants of repeat induced abortion among women seeking abortion care services at Debre Markos town health institutions, Amhara regional state, Ethiopia, 2017.

**Result:**

From the total 567-sample size, 547 women were participated in the study making a response rate of 96.5%. In this study 191 women reported that they had at least one previous abortion, making the proportion of repeat induced abortion 34.9%. In multivariable logistic regression analysis; illiteracy (AOR = 8.45, 95% CI 1.85, 36.49), living in an urban area (AOR = 5.14, 95% CI 2.29, 11.53), having multiple sexual partner (AOR = 6.16, 95% CI 3.25, 11.68), consuming alcohol (AOR = 2.77, 95% CI 1.52, 5.05) and having a history of physical violence by a male partner (AOR = 2.68, 95% CI 1.45, 4.94) were significantly associated with repeat induced abortion at p value less than 0.05.

## Introduction

Induced abortion (IA) is a deliberate surgical or medical termination of pregnancy that has not reached viability and includes both safe and unsafe abortions [1].

Globally, the annual estimation of IA that occurred from 2010 to 2014 was 56 million. Fifteen percent (8.3 million) of this IAs had occurred In Africa, and 2.7 million IA had occurred in East Africa. In 2014, there was an estimated number of 620,300 IA in Ethiopia, with an annual rate of 28 abortions per 1000 reproductive age women [2–5]. RIA accounts for a significant proportion of all IA in many countries, with reports ranging from 16 to 71% [1, 6]. In Ethiopia it accounts for 30 to 34 percent of all IA [7–9].

Termination of pregnancy in the first and second trimesters is associated with an increased risk of subsequent preterm birth, especially when performed with mechanical dilation and curettage or when performed repeatedly. Moreover, mid trimester pregnancy loss is more common in women who have had two or more induced or spontaneous abortions [10].

In Ethiopia, the number of women receiving treatment for complications from an abortion performed outside as well as inside a health institution increased between 2008 and 2014, rising from 52,600 to 103,600 [5].

Several measures have tried to prevent the impact of IA in Ethiopia. The major measure taken was providing post abortion care (PAC) as a response to avert deaths and injuries from abortion complications. Post abortion family planning (PAFP), a key component of PAC, has been provided specifically targeting to prevent repeat unintended pregnancies and repeat safe and unsafe abortions [11]. The International Conference on Population and Development (ICPD) Program of Action states that, “In no case should abortion be promoted as a method of family planning (FP)” [12]. Nevertheless, facts on unintended pregnancy and abortion in Ethiopia states that younger women who want to space births are using IA [13].

Despite the efforts to improve FP access and PAC specifically PAFP, the higher magnitude of repeat abortion in Ethiopia is worrisome. Furthermore, there is no study done on RIA and associated factor in Amhara region. Even if there are few studies done in Addis Ababa the capital city of Ethiopia, the available knowledge is insufficient to answer the relation of partner physical violence and substance exposure with RIA. Therefore, this study aimed to assess the magnitude and associated factors of RIA in Debre Markos town, Amhara region, Ethiopia.

## Main text

### Methods

#### Study design and period

An institutional based quantitative cross sectional study was conducted from October 1 to November 30, 2017 GC at health facilities of Debre Markos town, Amhara region, Ethiopia.

#### Source and study population

All reproductive aged women who seek abortion care services at health institutions of Debre Markos town were the study population, and those who seek the service at the selected health institutions of the town, during the study period, were the study population. Women on therapeutic abortion or those who had spontaneous abortion and came to the institution for PAC service were assumed to be excluded from the study.

#### Sample size and sampling procedure

The final sample size, 567, was calculated using single population proportion formula with the assumptions of 95% confidence interval, 5% margin of error, 33.6% magnitude of RIA in Addis Ababa from a previous study [9], 10% non-response and a design effect of 1.5. Multi stage sampling was the technique used to select the study participants. First, by using cluster sampling technique, health facilities with safe abortion service were clustered based on facility ownership as public, non-governmental and private facilities. There were five public health facilities (four public health centers and one public referral hospital), two facilities owned by Non-Governmental Organizations (NGO) and six private clinics in the town. By simple random sampling technique, two public, one NGO and four private health facilities were selected from each cluster. After allocating proportional sample for each cluster, based on their average monthly abortion service, systematic random sampling technique was finally employed to select the study participants from each health institution. The sampling interval K was calculated by using the formula K = N/n, and every second women was selected from each facility.

#### Data collection tool and procedures

The data was collected using an interviewer-administered questionnaire. It was conducted by eight diploma midwifes and supervised by four first degree holder midwifes. Prior to the data collection, proper training about the instrument and how to get consent for interview was given to the data collectors and supervisors for 3 days. The data collection tool was prepared from literatures, and first it was translated from English to the native language of the region (Amharic), and then re-translated to English language to ensure consistency. Moreover, pretest was conducted on 28 women who came for safe abortion service at University of Gondar referral hospital, Gondar town. Thereby, relevant modification was done to ensure the relevancy of the instrument before the actual data collection. The questionnaire contains; Part I—socio-demographic characteristics, Part II—sexual and reproductive health history, Part III—FP use and fertility intentions, Part IV—substance exposure status and Part V—gender based violence.

#### Operational definition

Repeat induced abortion—refers for the women who is having pregnancy termination for more than one time, without any medical or surgical indication.

#### Statistical analysis

First, the data was coded, entered and cleaned using Epi info statistical software version 7. Then, it was exported to SPSS statistical software version 20 for analysis. Descriptive statistical analysis such as simple frequencies, measures of central tendency and measures of variability were used to describe the characteristics of participants. To see the association between each independent variable with the outcome variable, bivariate logistic regression was used, and variables with a p value < 0.2 were selected for multivariable logistic regression. Independent predictors of RIA were determined by Multivariable logistic regression, and crude and adjusted odds ratios (COR & AOR) together with their corresponding 95% confidence intervals were computed to see the strength of association. Finally, level of statistical significance was declared at p-value < 0.05.

## Result

### Socio-demographic characteristics

Out of the total 567 sample size, 547 women were participated in the study making a response rate of 96.5%. The mean age of the participants was 23.98 (± 4.27) years and 42.6% of the participants were in the age group of 20–24 years. Half of the participants had an educational level of more than secondary (50.1%) and the majority (82.6%) was urban residents (Table 1).

**Table 1:** Socio-demographic characteristics and substance exposure status of women who seek abortion care services at Debremarkos town health institutions, 2017 (N = 547)

Variables	Frequency	Percentage
Age		
15–19	88	16.1
20–24	233	42.6
25–29	186	34.0
30–34	40	7.3
Religion		
Orthodox	490	89.6
Muslim	5	0.9
Protestant	52	9.5
Ethnic group		
Amhara	527	96.3
Oromo	12	2.2
Tigrie	8	1.5
Place of residence		
Urban	452	82.6
Rural	95	17.4
Level of education		
No education	46	8.4
Primary	80	14.6
Secondary	147	26.9
More than secondary	274	50.1
Marital status		
Married	61	11.2
Single	404	73.8
Divorced/separated/widowed	82	15.0
Occupation		
Unemployed	67	12.2
Student	157	28.7
Daily laborer	160	29.3
Merchant	71	13.0
Other	92	16.8
Total monthly income		
< 500	201	37.9
501–1000	230	43.4
1001–2000	70	13.2
2001–3000	25	4.7
> 3001	4	0.8
Ever smoke cigarette		
Yes	3	0.6
No	544	99.4
Ever chew khat		
Yes	10	1.8
No	537	98.2
Ever drink alcohol		
Yes	337	61.6
No [1]	210	38.4

### Sexual and reproductive characteristics

In this study, 191 women (34.9%) had RIA (95% CI (30.7–38)), and the majority (91.6%) had only one previous abortion. From the total study participants, most of them (78.6%) had their pregnancy unplanned and unwanted. Only 31.8% of the participants had gave birth previously. Over half (60.1%) received a PAFP method before they left the facility, and 47.2% and 72.5% of the participants reported a history of physical and sexual violence by their male partners, respectively (Table 2).

**Table 2:** Sexual and reproductive health characteristics of women who seek abortion care services at Debre Markos town health institutions, 2017 (N = 547)

Variables	Frequency	Percentage
Had multiple sexual partner		
Yes	245	44.8
No	302	55.2
Total no of past pregnancies		
0	251	46
1	167	30.5
2	62	11.3
≥ 3	67	12.2
Ever gave birth		
Yes	174	31.8
No	373	68.2
Total no of living biological children		
0	373	68.2
1	93	17.0
2	42	7.7
≥ 3	39	7.1
History of abortion		
Yes	191	34.9
No	356	65.1
Number of previous abortions (n = 191)		
1	175	91.6
2	16	8.4
Termination method of previous abortion (n = 191)		
Medication	150	78.5
Surgically	41	21.5
Ever use FP method		
Yes	272	49.7
No	275	50.3
Current FP use		
Yes	140	25.6
No	407	74.4
Receive FP counseling during this visit		
Yes	546	99.8
No	1	0.2
Desire for children in the future		
Yes	453	82.8
No	94	17.2
History of physical violence by male partner		
Yes	258	47.2
No	289	52.8
History of sexual violence by male partner		
Yes	395	72.2
No	152	27.8

### Factors associated with RIA

Five variables were significantly associated with RIA on multivariable logistic regression. Educational status had an association with the outcome variable. Participants with no education (AOR = 8.45, 95% CI 1.85, 36.49), with primary educational level (AOR = 5.46, 95% CI 2.06, 14.47) and with secondary educational level (AOR = 12.96, 95% CI 6.16, 27.29) were 1.85, 5.46 and 12.96 times more likely to have RIA compared to those who were above secondary educational level. Compared to rural residents, Woman who were urban residents, had 5.14 times chance of RIA (AOR = 5.14, 95% CI 2.29, 11.53). Woman who had multiple sexual partner were 6.16 times more likely to have RIA compared to their counter parts (AOR = 6.16, 95% CI 3.25, 11.68). Alcohol consuming woman had 2.77 times more chance to experience RIA compared to non-users (AOR = 2.77, 95% CI 1.52, 5.05). Woman who had physical violence by a male partner had 2.68 times more chance of engaging in RIA than those who had not (AOR = 2.68, 95% CI 1.45, 4.94) (Table 3).

**Table 3:** Bivariate and multivariable logistic regression analysis of determinants of repeat induced abortion among women who seek abortion care services at Debre Markos town health institutions, 2017 (N = 547)

Variables	Repeat abortion		COR (95% CI)	AOR (95% CI)
	Yes (%)	No (%)		
Place of residence				
Urban	172 (90.1)	280 (78.7)	2.457 (1.436, 4.205)	5.141 (2.291, 11.536)**
Rural [1]	19 (9.9)	76 (21.3)	1	1
Level of education				
No education	15 (7.9)	31 (8.7)	1.153 (0.591, 2.250)	8.452 (1.856, 36.491)*
Primary	25 (13.1)	55 (15.4)	1.083 (0.632, 1.857)	5.462 (2.061, 14.478)*
Secondary	70 (36.6)	77 (21.6)	2.166 (1.431, 3.280)	12.966 (6.160, 27.29)**
More than secondary	81 (42.4)	193 (54.2)	1	1
Had multiple sexual partner				
Yes	126 (66.0)	119 (33.4)	3.861 (2.662, 5.598)	6.162 (3.251, 11, 680)**
No [1]	65 (34.0)	237 (66.6)	1	1
Ever gave birth				
Yes	70 (36.6)	104 (29.2)	1	1
No [1]	121 (63.4)	252 (70.8)	0.713 (0.491, 1.035)	–
Ever use FP method				
Yes	114 (59.7)	158 (44.4)	1	1
No [1]	77 (40.3)	198 (55.6)	0.539 (0.377, 0.770)	–
Current FP use				
Yes	59 (30.9)	81 (22.8)	1	1
No [1]	132 (69.1)	275 (77.2)	0.659 (0.444, 0.978)	–
Ever drink alcohol				
Yes	149 (78.0)	188 (52.8)	3.17 (2.124, 4.733)	2.774 (1.523, 5.051)*
No [1]	42 (22.0)	168 (47.2)	1	1
Physical violence				
Yes	120(62.8)	138 (38.8)	2.67 (1.858, 3.837)	2.680 (1.453, 4.943)*
No [1]	71 (37.2)	218 (61.2)	1	1
Sexual violence				
Yes	152 (80.0)	243 (68.5)	1.844 (1.211, 2.806)	–
No [1]	38 (20.0)	112 (31.5)	1	1

1 Reference category

*Variables which are significantly associated with RIA (p-value < 0.05). ** Variables which are significantly associated with RIA (p-value < 0.001)

## Discussion

The overall magnitude of RIA for this study was 34.9% (95% CI (30.7–38.8)), the result is found to be comparable with two studies conducted at public and Non-governmental health institutions of Addis Ababa city, Ethiopia, 31% and 33.6% [8, 9].

The figure however is slightly higher than a study done in Kenya and Nigeria with magnitude of RIA 16% and 23% respectively [1, 14]. This might be due to the liberalized abortion law in Ethiopia may encourage women to seek for abortion care in a health institution. On the contrary, this study has smaller magnitude compared to the study done in Tunisia (42.2%) [15]. This may be due to the long time history of the liberalization of abortion law in Tunisia. Furthermore, this study has a much lower figure compared to two studies conducted in United States of America (USA) in New York (57%) and San Francisco (59%), [16, 17]. This may be due to the relatively better development stage of the country may contribute to better reporting of previous abortions.

Our study has identified that participants who had no education and had an educational level of primary and secondary were more likely to undergo a RIA than those who had an educational level of more than secondary. The finding is in consistent with the study in Ethiopia, Kenya, Tunisia, Georgia, and Russia [1, 8, 15, 18, 19]. Unplanned pregnancy secondary to poor contraceptive knowledge and use among those with a lower educational level might be the possible reason.

This study has found out urban residents had five time higher risk of having RIA than rural residents. This finding is consistent with the study done in Kenya [20]. This may be due to the low institutional service utilization of woman from rural area, and rather than seeking for safe abortion, they may choose to use unsafe abortion to terminate the pregnancy that reduces the figure.

In this study, woman who had multiple sexual partner were 2.68 times more likely to have a RIA than those who did not. This finding is consistent with the study done in Addis Ababa, Ethiopia and in Britain [8, 21]. This could be due to the fact that having multiple sexual partners will make those women to be in an unstable relationship, which leads to irregular use of contraceptive that will cause contraceptive failure and unwanted pregnancy.

This study has identified an association between alcohol use and RIA. Those who were consuming alcohol had a 2.7 times higher risk of having a RIA. This finding is consistent with the research done in San Francisco General Hospital, USA and with the study done in Russia [17, 19]. This may be because of the impact of alcohol on logical thinking of women and might lead them to have unprotected sex.

Finally, those who had a history of physical violence by a male partner had a 2.6 times higher risk of having a RIA than those who did not have a history of physical violence. This result is consistent with the studies done in Tunisia [15]. This could be due to fear of telling her male partner about the pregnancy and tend to protect herself by having an induced abortion.

## Conclusion

In nutshell, this study revealed a high rate of RIA in Debre Markos town health institutions. Upon the identified factors, it is required from health programmers and different NGOs to create awareness about the impact of RIA among those who are living in urban areas, has multiple sexual partner and less educated and students through mass media. Moreover, consider providing a youth friendly FP service at school clinics including primary and secondary schools. Rehabilitation centers and social support groups need to be widely accessible for alcohol users.

## Limitation

Due to the sensitivity of this issue, participants may tend to under report history of past abortion.

## Data Availability

To keep patients’ confidentiality, the raw data would not be shared. But, it is available from the corresponding author on reasonable request and the summary data are available in the main document.

## Abbreviations

AOR: adjusted odds ratio
CI: confidence interval
COR: crud odds ratio
Epi info: statistical package for epidemiological information analysis
FP: family planning
GC: Gregorian calendar
IA: induced abortion
ICPD: International Conference on Population and Development
NGO: Non-Governmental Organization
PAC: post abortion care
PAFP: post abortion family planning
PI: principal investigator
RIA: repeat induced abortion
SPSS: statistical package for social science
USA: United States of America
